# Measuring the effects of nurse-led frailty intervention on community-dwelling older people in Ethiopia: a quasi-experimental study

**DOI:** 10.1186/s12877-024-04909-2

**Published:** 2024-04-30

**Authors:** Ayele Semachew Kasa, Victoria Traynor, Peta Drury

**Affiliations:** 1 School of Nursing, Faculty of Science, Medicine, and Health, University of Wollongong, Wollongong (UOW), NSW Australia; 2 Department of Adult Health Nursing, College of Medicine and Health Sciences, Bahir Dar University, Bahir Dar, Ethiopia

**Keywords:** Frailty, Aged, Intervention, Effects, Nurse, Community, Ethiopia

## Abstract

**Background:**

Despite the critical need, interventions aimed at frailty in sub-Saharan Africa are scarce, attributed to factors such as insufficient healthcare infrastructure, the pressing need to address infectious diseases, maternal and child health issues, and a general lack of awareness. Hence, the aim of this research was to develop, implement, and evaluate the effect of a nurse-led program on frailty and associated health outcomes in community-dwelling older individuals in Ethiopia.

**Methods:**

This study utilised a pre-test, post-test, and follow-up single-group quasi-experimental design. The main outcome measure was to determine changes in the frailty levels of older individuals living in communities at three different intervals: initially (T0), immediately after the intervention (T1), and 12 weeks following the intervention (T2). Secondary outcomes were the observed changes in daily living activities, nutritional status, depression levels, and quality of life (QOL), evaluated at each of these data collection points. To analyse changes in frailty and response variables over these periods, Friedman’s ANOVA and Cochran’s Q test were employed, setting the threshold for statistical significance at *P* < 0.05.

**Results:**

Sixty-six older people with a high adherence rate of 97% completed the intervention and the follow-up measurements. Participants had an average age of 66.7 ± 7.9 years, with females comprising 79.4% of the group. Notably, 12 weeks post-intervention, there was a marked decrease in frailty (χ^2^(2) = 101.05, *p* < 0.001) and depression scores (χ^2^(2) = 9.55, *p* = 0.008) compared to the baseline. However, the changes in depression, physical, mental, and environmental domains of QOL were not sustained for 12 weeks post-intervention. Study participants showed an improvement in nutritional status (χ^2^(2) = 25.68, *p* < 0.001), activity of daily living (χ^2^(2) = 6.00, *p* = 0.05), and global quality of life (χ^2^(2) = 20.64, *p* < 0.001).

**Conclusions:**

The nurse-led intervention notably, 12 weeks post-intervention reduced frailty and depression. The intervention improved the nutritional status and some components of the quality of life of the participants. There is a need for further studies, especially with larger participant groups and stronger research designs such as randomized controlled trials (RCTs).

**Trial registration:**

ClinicalTrials.gov: NCT05754398 (03/03/2023).

## Background

The global population of individuals aged 60 or older is projected to surpass two billion by 2050, a significant increase from the 900 million recorded in 2015 [1]. Furthermore, worldwide estimates suggest that by 2030, one out of every six people will be in the 60-and-above age group [2]. The proportion of older people in low- and middle-income countries is expected to rise to 80% by 2050 [3], with Africa experiencing the most rapid growth in this demographic [4]. According to a 2017 United Nations (UN) report on aging populations, Africa’s older population is anticipated to grow from 69 million in 2017 to 225 million by 2050 [5]. This ageing trend places older people in Africa at a heightened risk of experiencing frailty [6].

Frailty is one of the most challenging aspects of ageing [7]. It is a complex and multifactorial condition commonly associated with increased vulnerability and a decline in physical, psychological, and social well-being, which results in a higher risk of adverse health outcomes such as falls, disability, institutionalisation, and mortality [8, 9]. Older people with frailty had a higher risk of hospital-associated costs [10]. Frailty can decrease social interaction, as older people may become isolated due to mobility issues or other health concerns [11]. This can lead to feelings of loneliness and depression, which can further impact quality of life (QOL) [12]. Frailty’s influence on the quality of life is especially pronounced in economically disadvantaged nations, including those situated in sub-Saharan Africa (SSA) [13, 14]. This can be attributed to various factors, which encompass inadequate nutrition, elevated poverty rates, restricted healthcare accessibility, and disparities in social conditions [15].

Even though the population is aging in low-income settings [6], research concerning ageing and age-related problems such as frailty is dominated by studies conducted in high-income settings [16]. According to various studies, the burden of frailty among community-dwelling older people is rising [16–18]. It appears to increase with age and be more prevalent in people with lower education and income, poorer health, and higher rates of comorbid chronic disease and disabilities [9]. In SSA, as life expectancy increases and the population ages, the number of frail older people is expected to rise [19, 20]. Many older people in this region suffer from chronic diseases such as Human Immunodeficiency Virus and Acquired Immune Deficiency Syndrome (HIV/AIDS), malaria, and tuberculosis, which can exacerbate frailty and lead to further decline in physical functioning [21]. In spite of this, current healthcare systems in low-and middle income countries are not well equipped to handle the healthcare needs of older people in the region [22].

A large proportion of older people in Ethiopia, battling with the challenges of frailty, remain in their local communities [23]. In these areas, the primary source of care and support comes from community nurses, who play a pivotal role in their well-being. Recent research in Ethiopia revealed that a notable 39% of the senior residents in these community settings were identified as frail [24]. This high incidence of frailty among Ethiopia’s older people highlights an urgent requirement for interventions and support systems to improve their health and quality of life. This situation calls for strategic health policies and tailored care programs, particularly focusing on this vulnerable group, to address and mitigate the factors contributing to frailty. The role of community nurses becomes even more critical in this context, as they are often the frontline providers of health care and support to these older individuals, making their involvement crucial in any intervention strategies aimed at this demographic.

Studies investigating interventions to reduce frailty amongst community-dwelling older people found that physical, nutritional, and cognitive interventional approaches were effective in reversing frailty among community-dwelling older people [25–27]. However these studies have predominantly been led by general practitioners and physiotherapists and very few have been led by nurses [28–30]. As frontline healthcare providers, nurses have frequent and direct contact with older people and are in a unique position to identify health promotion needs and provide education, counseling, and support to improve health outcomes [31]. Nurse-led interventions tested outside of Ethiopia, have shown the potential to improve health outcomes and alleviate the burden on acute hospital services for frail older individuals residing in the community [32]. In SSA, nurses provide primary healthcare services and act as a bridge between healthcare institutions and the populations they serve [33]. In Ethiopia, community health nurses are the backbone of the primary healthcare system and are involved in implementing various government funded healthcare interventions [34, 35].

Frailty research in SSA takes into account the diverse cultural, social, economic, and environmental factors that are specific to the African context. Using a set of variables, this study is the first to use an interventional design to reduce frailty and associated health outcomes among the African older population. Up to this point, no nurse-led initiatives have been undertaken to address frailty in community-dwelling older people in Sub-Saharan Africa, particularly in Ethiopia. This study aims to develop, implement, and evaluate the impact of a nurse-led intervention on frailty and the quality of life (QOL) of community-dwelling older people in Ethiopia.

## Methods

### Study design and hypothesis

The study was conducted using a pre-, post-, and follow-up single group quasi-experimental design [36–38]. The primary outcome was the change in frailty status of community-dwelling older persons measured at three points in time; baseline (T0), immediately after the end of the intervention (T1), and at 12 weeks post-intervention (T2). We hypothesised that frail older people who received the nurse-led intervention had a reduced frailty score, including the physical, psychological, and social domains of frailty. Secondary outcomes included changes in the activities of daily living, nutritional status, level of depression, and QOL measured at each data collection time point.

### Setting and sample

The study was conducted in Bahir Dar, Ethiopia. Bahir Dar is the capital city of the regional state of Amhara in Ethiopia. Based on a survey conducted by the Bahir Dar City Labour and Social Affairs Administration Office in 2018, it revealed that there were over 3,300 older people in Bahir Dar City [39].

The study sample size was calculated using a priori computation of sample size using G* Power version 3.1.9.4 [40] with the assumption of a two-tailed test with an alpha value of 0.05, an effect size (f) of 0.5, and a power of 0.95. By considering a 10 to 20% [41, 42] withdrawal rate during the intervention, 68 study participants were required.

### Recruitment

A poster including the aim of the study, eligibility criteria, and benefits of participating in the study was distributed through health posts and community gatherings using the local language. Furthermore, a list of older people in the selected sub city was obtained from the household’s registration, which is listed with the city’s administration health office. Potential study participants were recruited by the Community Health Workers (CHWs). During the home visit, the CHW explained the aim of the study, undertook a screen to determine frailty status, and obtained consent to participate in the intervention.

Each participant undertook a baseline assessment before starting the nurse-led intervention after confirming eligibility, willingness, and receiving written informed consent. The baseline assessment included socio-demographics, health-related factors, frailty, nutrition, depression, social support, activities of daily living, and QOL.

### Eligibility

The year in which ‘old age’ commences is determined by place of birth and the formal cutoff point legislated in social policy for each country [43]. In Ethiopia, the cutoff point for old age is 60 years [44, 45]. Therefore, older people 60 years or above, whose frailty score was five or more as measured by the Tilburg Frailty Indicator Amharic Version (TFI-AM), and residing in Bahir Dar, Ethiopia, were included in the study. Participants were excluded if they were unable to communicate, had cognitive impairment, were bed-ridden, had been hospitalised with a known psychiatric problem within the past six months, or did not live in the study area during the study period.

### Intervention

The intervention was designed based on the Integral Conceptual Model of Frailty (ICMF) framework. This framework denotes that the physical, psychological, and social domains of health are key components to ensure the health of frail older people [46, 47].

A nurse-led education intervention handbook culturally contextualised to frailty management for older people was developed. The education intervention handbook was created to offer continuous guidance to participants following their in-person educational sessions. Additionally, it was intended to serve as an extra resource for community health workers involved in carrying out the intervention. The content of the training handbook was based on the multi-dimensional concept of frailty [48–50] and customised to the local setting. The training handbook was reviewed by Ethiopian community nurses with experience in community healthcare services, and it was accompanied by illustrative pictures. The training handbook was translated into the local language, Amharic, and reviewed by a bilingual expert from Bahir Dar University (BDU), Ethiopia. A booklet focusing on the intervention was disseminated to the study participants during the first session of the nurse-led intervention.

The intervention comprised six independent, interconnected education sessions on:

Ageing and age-related changes, healthy nutrition, physical activity, mental health, social interaction and support, and an overall discussion. In each session, the intervention providers described the training with learning objectives prior to the training, asked leading questions of the session, and at the end of each session, study participants were given a simple take-home message. Moreover, the study participants had the opportunity to reflect on ideas, ask questions, and discuss with the intervention providers.

One of the six intervention components was delivered each month. At the end of six months all six intervention components had been delivered. Each session lasts approximately 40 to 60 min. All the six sessions were delivered one-on-one and face-to-face to the family homes of older people living in the community. During the six months when the intervention was delivered, there was a fortnightly 5 to 10-minute follow-up phone call with participants to receive feedback about the intervention sessions and provide opportunistic counseling on the specific topics. The intervention was delivered by two CHWs under overseen by lead investigator candidate leading this study. A Community Health Worker (CHW) is a registered nurse who works in a health post and in the community where study participants live. At the end of each session, the CHWs reflect on how each participant undertakes their take-home message and commence the subsequent education session from the reflection. To reduce loss to follow-up (LTFU) and increase adherence rates to intervention, participants were encouraged and reminded by phone to attend upcoming sessions. The intervention and content of the sessions were in line with a study protocol published by the research team [51]. The study protocol is registered in the ClinicalTrials.gov registry: NCT05754398 (03/03/2023).

### Data collection

The data were collected through a face-to-face administered structured survey questionnaire. The following physical data were also collected at the same time: height, weight, calf circumference, and midarm circumference. To reduce assessor bias, CHWs were not involved in the data collection process. Instead, two professional nurses from Bahir Dar city were recruited for data collection. Data collectors were not involved in the provision of the intervention. They took a one-day training about the characteristics of measurement tools, types of data to be collected, and how the study participants should be approached ethically. The data were collected at three time points: preintervention (baseline: T0), immediately after the intervention (postintervention: T1), and 12 weeks postintervention (follow-up: T2). The data collection questionnaire comprises different sections (Table 1).

**Table 1:** Outcome variables, data collection tools, and descriptions, 2023

Outcome variables	Data Collection Tools	Description	Interpretation
Change in frailty status of community-dwelling older people	TFI-AM	• 15 self-reported questions, divided into three distinct domains. • Physical, psychological, and social domains are the three distinct domains that constitute the TFI-AM. • It has been cross-culturally adapted, validated, and tested for reliability for use in Ethiopia.	• Score ranges from 0 to 15. • Higher score indicates worse frailty. • Frailty considered present when TFI-AM score is ≥ 5.
Change in the nutritional status	MNA	• Developed specifically for use in older people. • Most appropriate nutrition screening tool for use in community-dwelling older people. • Widely used including in Ethiopia.	• Scores ranges from 0 to 30. • Higher score indicates improved nutritional status. • MNA score classifies nutritional status of older people as: o Malnourished (MNA Score < 17), o At risk of malnutrition (MNA Score 17 to 23.5) or o Normal nutritional status (MNA Score 24 to 30)
Recent appetite change to predict weigh loss	SNAQ	• It is a brief, valid, and reliable four-item survey tool.	• A SNAQ score ranges from a minimum of 4 to a maximum score of 20 points. • SNAQ < 14 points, indicates a significant risk of at least 5% weight loss within six months.
Change in the level of depression	GDS-15	• Geriatric Depression Scale (GDS-15) extensively tested and validated in low and middle-income countries and across cultures including in Ethiopia.	• Score ranges from 0 to 15. • Higher score indicates worse depression. • Depression considered present when GDS score is ≥ 5.
Changes in the activities of daily living	Katz-ADL	• It ranks adequacy of performance in the six functions of bathing, dressing, toileting, transferring, continence, and feeding. • The instrument’s total score ranges from 0 to 6: higher scores indicate better activities in daily living.	• The responses are scored as a ‘Yes’ or ‘No’ for independence in each of the six functions. • A score of 2 or less indicates severe functional impairment, 4 indicates moderate impairment, and 6 indicates full function in activities of daily living.
Change in quality of life (QOL).	WHOQOL-BREF	• Self-report questionnaire or interviewer-administered consisting of 26 questions categorised into four domains that are scored on a 5-point Likert scale. • Four domains: physical health (7 items), psychological health (6 items), social relationships (3 items), and environment (8 items) [40–42]. • Item 1: Perceived rated QOL. • Item 2: Satisfaction with health. • Two items do not correspond to a domain and provide a global assessment of quality of life.	• Two items from the physical domain and one item from the psychological domain negatively framed. o Items 3, 4 and 26 are reverse coded for analysis. • Higher score indicates higher the quality of life [51].

GDS-15: Geriatric Depression Scale-15, MNA: Mini Nutritional Assessment, SNAQ: Simplified Nutritional Appetite Questionnaire, TFI-AM: Tilburg Frailty Indicator-Amharic Version, WHOQOL-BREF: World Health Organization Quality of Life BREEF

#### Ethics

The study was approved by the University of Wollongong (UOW) Human Research Ethics Committee (HREC) with the approval number of 2022/212 on the 12th of September 2022 and by the Institutional Review Board (IRB) of College of Medicine and Health Sciences (CMHS) Bahir Dar University (BDU) with the approval number of 563/2022 on the 24th of October 2022.

### Data analysis

Study participants who received all the nurse-led intervention sessions were included in the final analysis to determine the effect of the nurse-led intervention to reduce frailty among older people in Ethiopia. The information collected through paper-based questionnaires were inputted into EpiData Manager software and then exported to IBM SPSS 26.0 (IBM Corp., Armonk, NY, USA) for analysis. To describe the data, frequency and percentage distributions of categorical data and the mean (± *SD*) of numerical data were summarised. At baseline, to measure the degree of association between TFI-AM score and QOL, a Spearman rank correlation was carried out. For variables having two, and three, or more categories, the Mann-Whitney U-Test and Kruskal Wallis test were used, respectively, to compare their mean scores on the overall frailty score.

To evaluate the effect of the nurse-led intervention, a one-way repeated measures ANOVA was planned. However, the assumptions of the analysis of variance did not fulfill some of the stringent assumptions of including the observations to be normally distributed across the three time points. As a result, the nonparametric alternative test, Friedman’s ANOVA was used. The Cochran’s Q test was used to evaluate changes in the proportion of participants screened with the outcome measures with dichotomous responses across the three time points. Kendall’s W values were presented as a measure of effect size. A value of 0.10 to < 0.30 (small effect), 0.30 to < 0.50 (moderate effect), and ≥ 0.50 (large effect) [52, 53]. The level of statistical significance was set at *P* < 0.05.

### Result

**Fig. 1:**
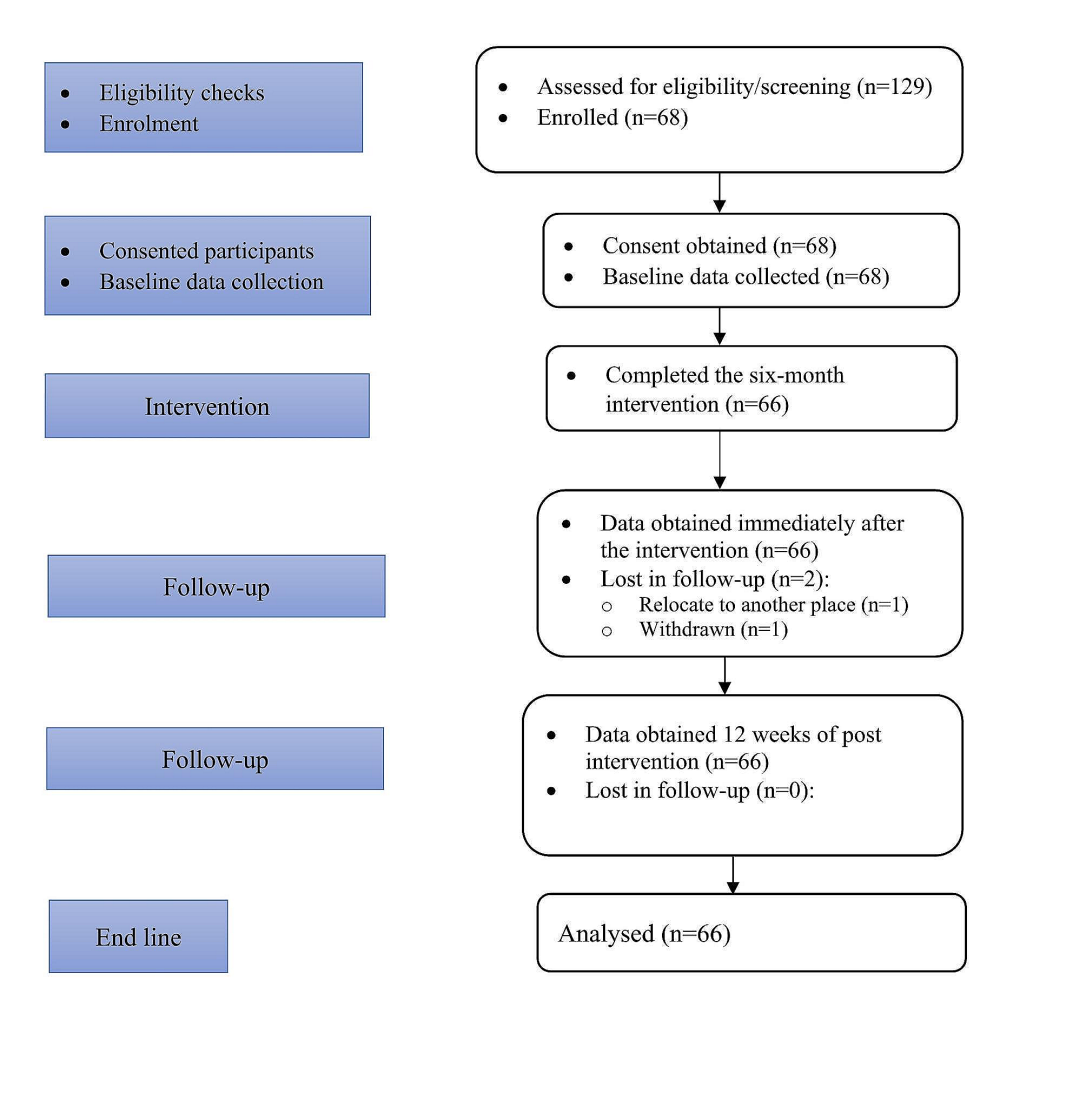
The flow chart of educational intervention of the study, 2023

Out of 129 older people assessed for eligibility, 68 were initially selected for the study. During the course of the study, data from two participants was not included in the final analysis: one participant withdrew from the study, while the other moved away from the study area. Consequently, 66 participants successfully completed both the intervention and the subsequent assessments with a high adherence rate of 97%. The final analysis, focused on evaluating the impact of the nurse-led intervention on reducing frailty in Ethiopia’s older population, included only those participants who attended all the intervention sessions and were measured at the three designated time points (Fig. 1).

### Study participant characteristics

The average age of participants in the study was 66.7 years, with a standard deviation of 7.94. A significant majority, 79.4%, were female. A notable 63.2% of the participants were unable to read and write. About 60% resided with their daughter or son, and a substantial 83.8% received caregiver support. Over half of the participants, 52.9%, sought health check-ups only when necessary. Around 44% were dealing with confirmed medical conditions, and more than half were enrolled in Community-Based Health Insurance. There were no significant differences observed in demographic variables related to overall frailty, except for living arrangements, H (2, *n* = 66) = 6.75, *p* = 0 03 and Community-Based Health Insurance usage (z = -2.00, *p* = 0.04). Educational status exhibited a marginally significant difference in relation to overall frailty (Table 2).

**Table 2:** Baseline socio-demographic and related characteristics (*n* = 66), 2023

Variables		n (%)	Median	P
Gender	Male	14 (20.6)	6.5	0.95
	Female	54 (79.4)	7.0	
Marital status	Married	22 (32.4)	6.0	0.24
	Divorced	18 (26.4)	7.0	
	Widowed	28 (41.2)	7.5	
Educational status	Not able to read and write	43 (63.2)	7.0	0.05
	Able to read and write	25 (36.8)	7.0	
Job status	Not working	31 (45.6)	8.0	0.11
	Retired	28 (41.2)	7.0	
	Other¥	9 (13.2)	7.0	
Living arrangement	With spouse	22 (32.4)	6.0	0.03
	With children	41 (60.3)	7.0	
	Alone	5 (7.3)	11.0	
Availability of caregiver support	Yes	57 (83.8)	7.0	0.68
	No	11 (16.2)	7.0	
Frequency of health check-ups	Only when needed	36 (52.9)	7.0	0.74
	Once in three months	26 (38.2)	7.0	
	Other*	6 (8.9)	8.0	
Living with medically confirmed health problem	Yes	30 (44.1)	7.0	0.57
	No	38 (55.9)	7.0	
Prescribed medication in the last 24 h	None	38 (55.9)	7.0	0.87
	One	14 (20.6)	6.5	
	Two and more	16 (23.5)	7.0	
History of a fall in the last one year	Yes	21 (30.9)	8.0	0.11
	No	47 (69.1)	7.0	
Use of CBHI	Yes	37 (54.4)	8.0	0.04
	No	31 (45.6)	7.0	
Nutritional status^(a)^	Malnourished, n (%)	14 (20.6)	9.0	< 0.01
	Risk of malnutrition, n (%)	34 (50.0)	7.0	
	Well-nourished, n (%)	20 (29.4)	6.0	
Recent appetite^(b)^	Risk for 5% weight loss within 6 months, n (%)	47 (69.1)	7.0	0.09
	No risk of weight loss, n (%)	21 (30.9)	7.0	
Depression status^(c)^	Depressed, n (%)	23 (33.8)	9.0	0.001
	Non-depressed, n (%)	45 (66.2)	7.0	
Activity of daily living^(d)^	Full functioning, n (%)	58 (85.3)	7.0	0.280
	Moderate impairment, n (%)	10 (14.7)	8.0	
Age (mean ± SD) in years		66.7 ± 7.9		

(a) Mini Nutritional Assessment, (b) Simplified Nutritional Appetite Questionnaire, (c) Geriatric depression rating scales−15, (d) Katz Index of Independence in Activities of Daily Living, CBHI: Community−Based Health Insurance, n: number of cases, P: level of significance, SD: Standard Deviation, ¥ Daily labourer, merchant

### Follow-up: outcome measures over time

The intervention was implemented for 24 weeks (6 months) between January and June 2023. Outcomes were measured pre-intervention (baseline: T0), immediately after the intervention (post-intervention: T1), and 12 weeks post-intervention (follow-up: T2). The data consisted of the responses of participants who completed the questionnaire at all data collection time points.

Improvements were detected in the results reported from baseline to post-intervention and follow-up. The results of the Friedman test indicated that there was a statistically significant difference in the overall frailty scores across the three-time points (pre-intervention (T0), immediately post-intervention (T1), and 12-weeks post-intervention (T2) χ^2^(2) = 101.05, *p* < 0.001). The changes were also observed across the frailty domains. However, it was not significant in the domain of social frailty χ^2^(2) = 2.93, *p* = 0.23). The test revealed a statistically significant difference in MNA score χ^2^(2) = 25.68, *p* < 0.001) and Geriatric Depression Scale-15 (GDS-15) score χ^2^(2) = 9.55, *p* = 0.008) across the three-time points. The test also revealed that there was a statistically significant difference in the quality-of-life domains. The finding revealed a statistically significant difference in the physical health of QOL domain score χ^2^(2) = 24.50, *p* < 0.001), and mental health of QOL domain score χ^2^(2) = 6.83, *p* = 0.033) across the three time points. The effects of the nurse-led intervention program were sustained for 12 weeks of post-intervention. However, the changes in depression, physical, mental, and environmental domains of QOL were not sustained for 12 weeks of post-intervention (Table 3).

**Table 3:** Changes in outcome measurements at three points in time (T0, T1, and T2) (*n* = 66), 2023

Outcome measures	Mean rank			F^a^	P	Effect size^b^
	Baseline (T0)	Post-intervention (T1)	Follow-up (T2)			
Overall frailty	2.93	1.60	1.47	101.05	< 0.001	0.76
Physical frailty	2.79	1.64	1.57	80.56	< 0.001	0.61
Psychological frailty	2.30	1.90	1.80	13.28	0.001	0.10
Social frailty	2.11	1.96	1.93	2.93	0.23	0.02
MNA	1.52	2.19	2.30	25.68	< 0.001	0.19
SNAQ	1.55	2.18	2.27	28.50	< 0.001	0.22
Katz ADL	1.93	2.00	2.07	6.00	0.05	0.04
GDS	2.27	1.82	1.91	9.55	0.008	0.07
WHOQOL-BREF						
Physical health	1.58	2.37	2.05	24.50	< 0.001	0.18
Mental health	2.04	2.18	1.78	6.83	0.033	0.05
Social relations	1.76	1.89	2.36	16.95	< 0.001	0.13
Environment	1.78	2.17	2.05	6.33	0.042	0.04
Global QoL	1.74	2.13	2.13	20.64	< 0.001	0.15
Global Health	1.99	1.99	2.03	0.22	0.89	< 0.01

^a^Friedmans ANOVA, ^b^Kendall’s w, ADL: Activity of daily living, GDS: Geriatric depression scale, MNA: Mini Nutritional Assessment, SNAQ: Simplified Nutritional Appetite Questionnaire

**Fig. 2:**
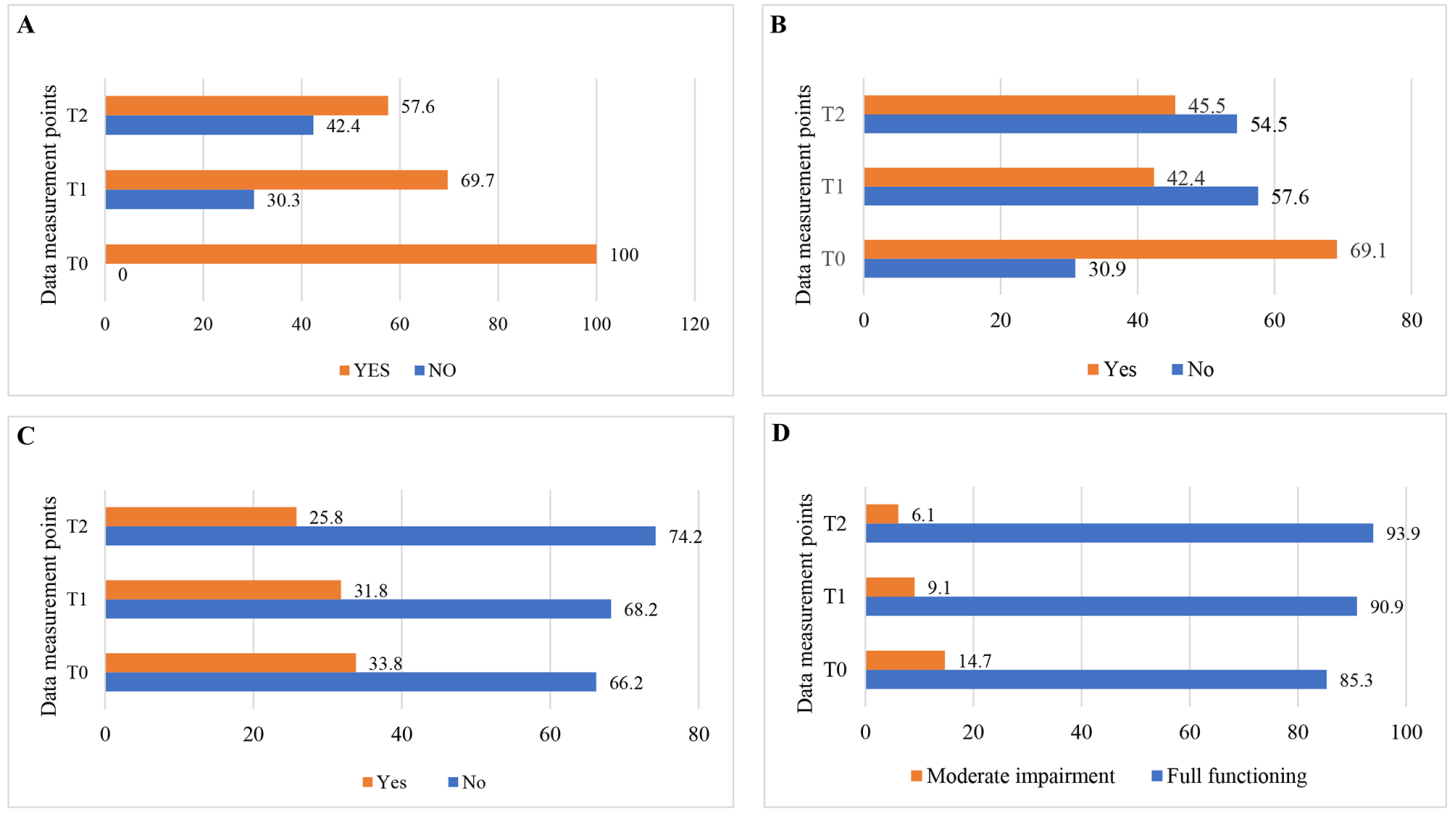
Percentage of change in frailty score **(A)**, risk of weight loss **(B)**, depressive symptoms **(C)**, and activity of daily living **(D)** measurements over time (*n* = 66), 2023

The Cochran’s Q test revealed that there was a significant change in the proportion of frailty across the three time points (χ^2^(2) = 40.25, *p* < 0.01) (Fig. 2a). The result revealed a significant reduction in the proportion of the risk of weight loss from 69.1% at T0 to 42.4% at T1. However, there was a slight increase in the risk of weight loss from T1 to T2 (χ^2^(2) = 16.18, *p* < 0.01) (Fig. 2b). There was a reduction in the proportion of depression from 33.8% at T0 to 25.8% at T2. However, Cochran’s Q test revealed that the changes were not significant (χ^2^(2) = 1.91, *p* = 0.38) (Fig. 2c). In the activity of daily living, there was a significant reduction in the proportion of moderate impairment from 14.7% at T0 to 6.1% at T2 (χ^2^(2) = 44.27, *p* < 0.01) (Fig. 2d).

## Discussion

This study aimed to design, implement, and evaluate the effects of a nurse-led intervention on frailty. To the best of our knowledge, this was the first intervention trial that examined the effect of a nurse-led intervention using a multidimensional frailty measurement instrument in sub-Saharan Africa and more specifically in Ethiopia. To promote successful implementation, a culturally appropriate and contextually relevant frailty intervention handbook was designed based on the ICMF framework [46, 47]. This framework highlights the importance of addressing the physical, psychological, and social domains of frailty as crucial elements in maintaining the well-being of frail older people [54].

This research highlights that a nurse-led, multifaceted intervention delivered in the community can mitigate frailty and improve frailty health outcomes. The success of the intervention was evident by a reduction in average frailty scores from the initial assessment to six months and 12 weeks after the intervention, from 2.93 to 1.60 and 1.47, respectively. These findings align with a previous studies that which have reported a consistent decrease in average frailty scores from the baseline to the follow-up period [48, 55, 56]. Additionally, our study corroborates other research indicating that exercise interventions, encompassing endurance, strength, coordination, balance, and flexibility exercises, have successfully reversed frailty in older people with such conditions [57–59]. The prevailing consensus in existing literature suggests that long-term, multicomponent interventions are particularly beneficial for frail older adults, which is also supported by our study. Specifically, interventions that include resistance or balance exercises, flexibility activities, and nutritional components are found to be most effective for this demographic [60].

Frailty encompasses impairments in physical, psychological, and social functions [8, 9], and the results of our study demonstrate that it is possible to enhance physical and psychosocial functioning through a multidimensional intervention that targets these contributing factors [48]. Prior studies investigating multicomponent interventions, which address multiple underlying causes of frailty such as muscle weakness, malnutrition, and psychosocial symptoms, have been shown to be significantly more effective in reversing frailty in older people compared to single interventions [61]. Furthermore, these interventions not only contributed to improved physical function but also enhance overall health [62]. In the current study, the nurse-led intervention reversed the physical, psychological, and social domains of frailty. However, the improvements in the social frailty domain were not significantly sustained over 12 weeks of follow-up. This may be due to the complexity and multifaceted nature of social frailty, which can be influenced by various factors such as social support, community engagement, and culture, making it challenging to identify meaningful changes in this domain over time [63].

In this study, there was a notable rise in Mini Nutritional Assessment scores, with this improvement persisting throughout a 12-week post-intervention period. This aligns with earlier research that also documented a significant enhancement in MNA scores [42]. Other studies have indicated that nutritional interventions and education play a vital role in boosting physical functioning and psychosocial health, which in turn contributes to a reduction in frailty [64]. Enhanced nutritional status coupled with increased physical activity leads to improved appetite and stronger muscle strength and improved overall wellbeing. Such improvements are beneficial for increasing strength and functional capacity, thereby contributing to the reversal of frailty [65].

The nurse-led, multicomponent intervention in this study led to a significant change in depression levels, as evidenced by a notable decrease in GDS-15 scores from the baseline to the six-month assessment. This aligns with previous research where a similar intervention was shown to reduce depressive symptoms [66]. However, unlike findings in another study where the effects of a nurse-led multicomponent program were sustained over 12 weeks in the experimental group [42, 48]. the reduction in depressive symptoms in our study did not maintain its trajectory for 12 weeks of follow-up. This discrepancy may be due to differences in how the intervention was administered. In contrast to our approach, the use of group-based activities, shorter follow-up periods with more frequent intervention sessions, and the inclusion of music to enhance enjoyment could improve the long-term effectiveness in reducing depression by addressing both social and emotional aspects. The success of such interventions, however, can vary across different cultures and individuals, highlighting the need for customisation to cater to the specific needs and cultural expectations of each participant [67].

At the six-month mark, there was a statistically significant enhancement in all quality-of-life domains among participants, when compared to baseline measurements. This outcome echoes findings from another study where QOL notably improved following the implementation of an intervention to reduce frailty [65]. Additionally, research has shown that multicomponent, nurse-led interventions specifically designed for frail older adults can lead to more pronounced improvements in QOL [41]. However, in this study, these improvements were not consistently maintained across most QOL domains during the 12-week follow-up period. This contrasts with observations that frail older people might need continuous support addressing their medical, emotional, and financial needs to sustain their quality of life over time [68].

The effect of our nurse-led intervention was assessed using one of the multidimensional frailty assessment instruments, the TFI-AM, following cross-cultural adaptation, validity, and reliability testing [69]. In low-income countries, multidimensional frailty assessments will play an important role in providing a comprehensive assessment of health status, facilitating individualised care, and informing public health planning. However, the high heterogeneity observed in frailty measurement instruments across studies makes it difficult to discuss the effect of the intervention when varying frailty measurement tools have been adopted. As a result, multicomponent and standardised instruments are needed to ensure comparability and facilitate the synthesis of meaningful findings.

Our nurse-led frailty intervention resulted in significant reduction in frailty. However, ongoing physical and psychosocial support to community-dwelling frail older people is needed to meet their physical, emotional, and social needs to sustain these changes over time. Our findings have contributed to the limited data on the effect of a nurse-led frailty intervention in sub-Saharan Africa and more specifically in Ethiopia. In most sub-Saharan African regions, primary healthcare professionals are not sufficiently trained to identify, manage, and evaluate many geriatric syndromes, including frailty [70]. Therefore, the need to improve the existing primary healthcare system in the region more specifically in Ethiopia, targeting training of primary healthcare professionals in designing person-centred care for older people.

### Strengths and limitations

The strength of this study was the development, implementation and evaluation of a nurse-led intervention based on a pre-designed protocol [51]. A quasi-experimental design was adopted to determine the effect of the intervention delivered one-on-one and face-to-face to the family homes of frail older people living in the community. Quasi-experimental (non-randomised) studies are increasingly adopted to evaluate population health interventions by health experts [71, 72]. Achieving high adherence rate the study participants in completing the intervention was another strength of the study. To our knowledge, this is the first study evaluating the effect of nurse-led frailty interventions based on the ICMF among older people not only in Ethiopia but also in in SSA regions. Moreover, such interventions are likely to be replicable since there is a handbook that is specifically designed for frailty intervention. However, there are some limitations. The current intervention may be limited by the lack of a control group, which will make it difficult to determine whether changes over time are due to the intervention or other external factors. Lack of randomisation will introduce selection bias and confounding, impacting the internal validity of the study. The lack of improvement observed on the quality-of-life questionnaires may be linked to social desirability bias. The other limitation is a relatively small sample size. Study participants were recruited from one region in Ethiopia resulting in an unrepresentative result. Despite the priori power analysis showed that our sample was adequate, the relatively small sample size leaves our study more susceptible to potential confounding effects and will limit the generalisability of the findings. Future research is needed to increase the sample size and confirm the generalisability in different settings and contexts.

## Conclusions

It is inevitable that there will be an increase in frail older persons in sub-Saharan Africa, such as in Ethiopia, which has the second largest older population in Africa. The results indicated nurse-led frailty interventions with a variety of components in community-dwelling older people demonstrated frailty reversal, a reduction in depressive symptoms, and an improvement in nutritional status and quality of life. It is essential that frail older people are assisted to live as safely and independently as possible in the community. However, research using larger samples and more rigorous study designs, such as randomised controlled trials (RCTs), in the future would be needed. Moreover, a pragmatic mixed design could also be beneficial for future research. To effectively address the multifactorial nature of frailty, nursing-led interventions must be ongoing and comprehensive to effectively sustain the required changes over time. The World Health Organisation and UN urged to fund intervention studies in the ageing sector in the SSA in order to address the unique needs of older people, promote healthy ageing, generate evidence for policymaking, and build research capacity. Investing in such programs can result in improved health outcomes, enhanced well-being, and sustainable development in the face of the ageing demographic transition occurring in Africa. This will in turn contribute for the attainment of Sustainable Development Goal (SDG) 3 and 10 in the SSA region.

## Data Availability

All data generated or analysed during this study are included in this manuscript.

## Abbreviations

ADL: Activity of daily living.
BDU: Bahir Dar University.
CBHI: Community-Based Health Insurance.
CHWs: Community Health Workers.
CMHS: College of Medicine and Health Sciences.
GDS-15: Geriatric Depression Scale-15.
HREC: Human Research Ethics Committee.
ICMF: Integral Conceptual Model of Frailty.
IRB: Institutional Review Board.
MNA: Mini Nutritional Assessment.
QOL: Quality of life.
SD: Standard Deviation.
SSA: sub-Saharan Africa.
TFI: Tilburg Frailty Indicator.
TFI-AM: Tilburg Frailty Indicator Amharic Version.
UN: United Nations.
UOW: University of Wollongong.
